# Healthy Doctrine

**Published:** 1856-02

**Authors:** 


*Healthy Doctrine.*—The Medico-Chirurgical Review, (London,) has a lengthy notice of Meigs on Childbed Fever. We are pleased to notice that the reviewer, Dr. Fleetwood Churchill, subjects the work to a careful analysis, and differs with Dr. Meigs upon all the main issues. Dr. Churchill considers Childbed Fever as a disease distinct from the local inflammation, as contagious, as typhoid in its character, and as requiring stimulants rather than depletion. The Review, though kind in tone, is nevertheless caustic in fact and triumphant in argument. Dr. Meigs could hardly have written another work so dangerous to his reputation as a medical reasoner as this.

